# Syphilis reinfection is associated with an attenuated immune profile in the same individual: a prospective observational cohort study

**DOI:** 10.1186/s12879-018-3399-8

**Published:** 2018-09-25

**Authors:** Chris Kenyon, Kara Krista Osbak, Tania Crucitti, Luc Kestens

**Affiliations:** 1 HIV/STI Unit, Institute of Tropical Medicine, Antwerp, Belgium; 2 Division of Infectious Diseases and HIV Medicine, University of Cape Town, Anzio Road, Observatory, Cape Town, 7700 South Africa; 3 HIV/STI Reference Laboratory, Institute of Tropical Medicine, Antwerp, Belgium; 4 Immunology Unit, Institute of Tropical Medicine, Antwerp, Belgium; 5 Department of Biomedical Sciences, University of Antwerp, Antwerp, Belgium

**Keywords:** Syphilis, Immunology, Repeat, Screening, Cytokine

## Abstract

**Background:**

Ascertaining if the clinical and immunological response to repeat syphilis differs from that in initial syphilis may assist in designing optimal syphilis screening strategies and vaccine design.

**Methods:**

We prospectively recruited 120 patients with a new diagnosis of (baseline) syphilis. During a 24-month follow-up period, 11 of these patients had a further diagnosis of (repeat) syphilis. We conducted a paired comparison of their plasma cyto-chemokines at baseline and repeat syphilis.

**Results:**

Comparing to their baseline infection, paired analyses of the 11 individuals with repeat infections during follow-up revealed that these reinfections had lower concentrations of Interferon (IFN)α (0.8 [Interquartile range (IQR) 0.8–0.8 vs. 12.2 [IQR 1.6–24.2], *P* = 0.004) and Chemokine (C-C motif) ligand (CCL) 4 (0.9 [IQR 0.9–12.2 vs. 17.5 [IQR 4.9–32.8], *P* = 0.022].

**Conclusion:**

In this small study of 11 individuals, repeat syphilis was found to present with an attenuated immune response. The relevance of these findings to the design of optimal syphilis screening programs is discussed.

## Background

A striking feature of the current epidemics of syphilis in high-income countries is the increasing proportion of cases of syphilis that are repeat episodes of syphilis [1–3]. These infections occur predominantly in men who have sex with men (MSM) with high rates of partner change [1, 4]. There is growing recognition that part of the difficulty with controlling these epidemics is the fact that repeat syphilis is less likely to present with symptoms [4–8]. As a result, persons with repeat syphilis may have delayed diagnoses and be more likely to transmit syphilis [1, 6]. At our medical centre in Antwerp, Belgium, reinfections now constitute approximately half of all syphilis infections [1]. In a study that followed up 120 patients with a new diagnosis of syphilis over a 12-month period we previously confirmed that repeat syphilis was not only less likely to be symptomatic than initial syphilis but also had an altered immune profile [4]. These differences included variations in the magnitude and decay curves of rapid plasma reagin (RPR) as well as repeaters being less likely to have elevated plasma interleukin (IL)-10. These differences in plasma IL-10 persisted at 6-month follow-up such that those with initial but not those with repeat syphilis had higher levels of IL-10 than the control group. While following up this cohort for a 24-month period a total of 11 individuals had a further repeat episode of syphilis. This manuscript compares the immune responses of these 11 repeat episodes with the paired baseline episodes.

## Methods

The Institutional Review Board of the Institute of Tropical Medicine and the Ethics Committee of the University Hospital Antwerp approved this study (13/44/426). All study participants provided written informed consent. Between May 2014 and June 2015 all patients attending the Institute of Tropical Medicine, Antwerp’s STI or HIV clinics and over the age of 17 years in whom a new diagnosis of syphilis was made were prospectively recruited into the study (ClinicalTrials.gov Registration Number: NCT02059525). The diagnosis and staging of syphilis was according to the Centers for Disease Control and Prevention classification [9]. Repeat syphilis, which included syphilis repeat infections and reactivations, was defined as an episode in a person who had a ≥ 4 increase in RPR titre after a previous diagnosis of syphilis who exhibited an appropriate response to therapy, defined as a ≥ 4-fold decrease in RPR titre. All patients were assessed according to a standardized protocol that collected detailed information about sexual behavior, clinical features and laboratory tests. Patients were followed up at 3, 6, 9, 12, 18, 24 months and at any other time if they developed symptoms or signs of syphilis. Patient clinical and laboratory characteristics were recorded at each consultation. Blood was drawn into plain and EDTA-coated tubes (Sarstedt Monovette, Nümbrecht, Germany) and centrifuged at 2000 *g* for 10 min at ambient temperature. Serum and plasma was stored at − 20 °C (plain) and − 80 °C (plasma), respectively. Samples were processed and frozen within 3 h of acquisition.

Interferon (IFN)α, IFNγ, IL-1β, IL-12p40, IL-12p70, Interferon gamma inducible protein (IP)-10, Monocyte chemoattractant protein (MCP)-1, Macrophage inflammatory protein (MIP)-1α// Chemokine (C-C motif) ligand (CCL)3, CCL4 (MIP-1β), IL-4, IL-5, IL-6, IL-7, IL-8, IL-10 and IL-17A from the baseline, 6 month follow-up and repeat episodes of syphilis were measured in a single experiment using a magnetic bead Luminex multiplex assay (EMD Millipore, Billerica, MA, United States (US)) according to the manufacturer’s instructions. Samples below the limit of quantification were assigned the value of half the lowest limit detected for each cytokine (for further study details including quality assessment data please see [4]). Macro Vue RPR card test (Becton, Dickinson and Company, Sparks, MD, US) and *Treponema pallidum* agglutination chemiluminescent immunoassay (Ortho-Clinical Diagnostics, Rochester, NY, US) performed on the Vitros 5600 Integrated System (Ortho-Clinical Diagnostics) were performed at each study visit.

### *T. pallidum* PCR

An in-house real time PCR targeting *polA* was used to detect *T. pallidum* DNA in serum. DNA was extracted from 400 μL serum using the custom-plasma program of the Abbott m2000sp automated extraction platform (Abbott, Maidenhead, UK), according to the manufacturer’s specifications. The extracted DNA was eluated in 100 μL eluate solution. The 25 μL PCR mixture contained Platinum® Quantitative PCR SuperMix-Uracil DNA Glycosylase (Invitrogen by life technologies, Thermo Fisher Scientific) 0.9 μM forward primer TP-1, 0.9 μM reverse primer TP-2,0.14 μM probe TP-3 (Integrated DNA technologies, Leuven, Belgium) [10] and 10 μL DNA extract. The amplifications were performed using the Rotor Gene 6000 (Qiagen, Venlo, the Netherlands) and included 45 cycles of 20 s at 95 °C followed by 45 s at 60 °C per cycle.

### Statistical analysis

Values are summarized as medians and interquartile ranges (IQR). Wilcoxon’s signed rank test was used to evaluate intraindividual changes from baseline to repeat syphilis. Spearman’s correlation was used to assess if there was an association between the number of prior episodes of syphilis and plasma cytokine and chemokine concentrations at the time of syphilis diagnosis (baseline and repeat infections combined, *N* = 22). All analyses were performed in Stata 13 (StataCorp LP, College Station, TX, USA). Due to the small sample size we did not conduct multivariable analyses.

## Results

The cohort was followed up for 24 months. During this time period 11 individuals (all men, MSM and 10 HIV-infected) were diagnosed with a syphilis reinfection a median 456 (IQR 364–503) days after their baseline infection (Table 1). The median CD4+ T-cell count of the 10 HIV-infected individuals was 554/μL (IQR 489–677) at the baseline visit. All HIV-infected individuals were on antiretroviral therapy with undetectable HIV viral loads. Serum PCR for *Treponema pallidum* was negative for all 11 individuals at baseline and for 6/6 at the time of reinfection. All 11 were treated with stage appropriate intramuscular benzyl penicillin G (BPG) therapy at baseline and reinfection syphilis.

**Table 1:** Selected clinical and laboratory findings of 11 individuals with an episode of syphilis reinfection during study follow-up

		Baseline Episode of Syphilis						Nadir RPR	Repeat Episode of Syphilis		
Study nr	HIV Status	CD4 (T cells/ul)	HIV Viral Load	No. Previous Syphilis Infections^a^	Days since last syphilis	RPR titre baseline	Syphilis Stage	Nadir RPR pre- reinfection	RPR Repeat	Syphilis Stage	Days between baseline and repeat syphilis
1	Infected	563	< 20	3	1371	256	Secondary	64	256	Early Latent	391
2	Infected	546	< 20	4	692	128	Early Latent	16	256	Early Latent	532
3	Infected	677	< 20	1	360	64	Early Latent	8	128	Early Latent	456
4	Infected	456	< 20	1	347	256	Secondary	16	64	Early Latent	492
5	Infected	598	< 20	2	568	8	Early Latent	2	16	Primary	424
6	Uninfected	NA	NA	1	319	64	Secondary	2	8	Early Latent	725
7	Infected	489	< 20	6	684	128	Late Latent	16	64	Primary	496
8	Infected	1284	< 20	1	180	128	Secondary	8	32	Early Latent	308
9	Infected	689	< 20	1	797	128	Late Latent	32	256	Secondary	364
10	Infected	503	< 20	1	565	128	Secondary	4	256	Early Latent	503
11	Infected	483	< 20	0	NA	32	Secondary	0	32	Secondary	171

^a^ Number of episodes of syphilis prior to current study

*NA* Not applicable

### Syphilis stage at reinfection

Two of these reinfections were primary, two secondary and seven early latent syphilis (Table 1).

### Only one individual presented with confirmed initial syphilis

**Fig. 1:**
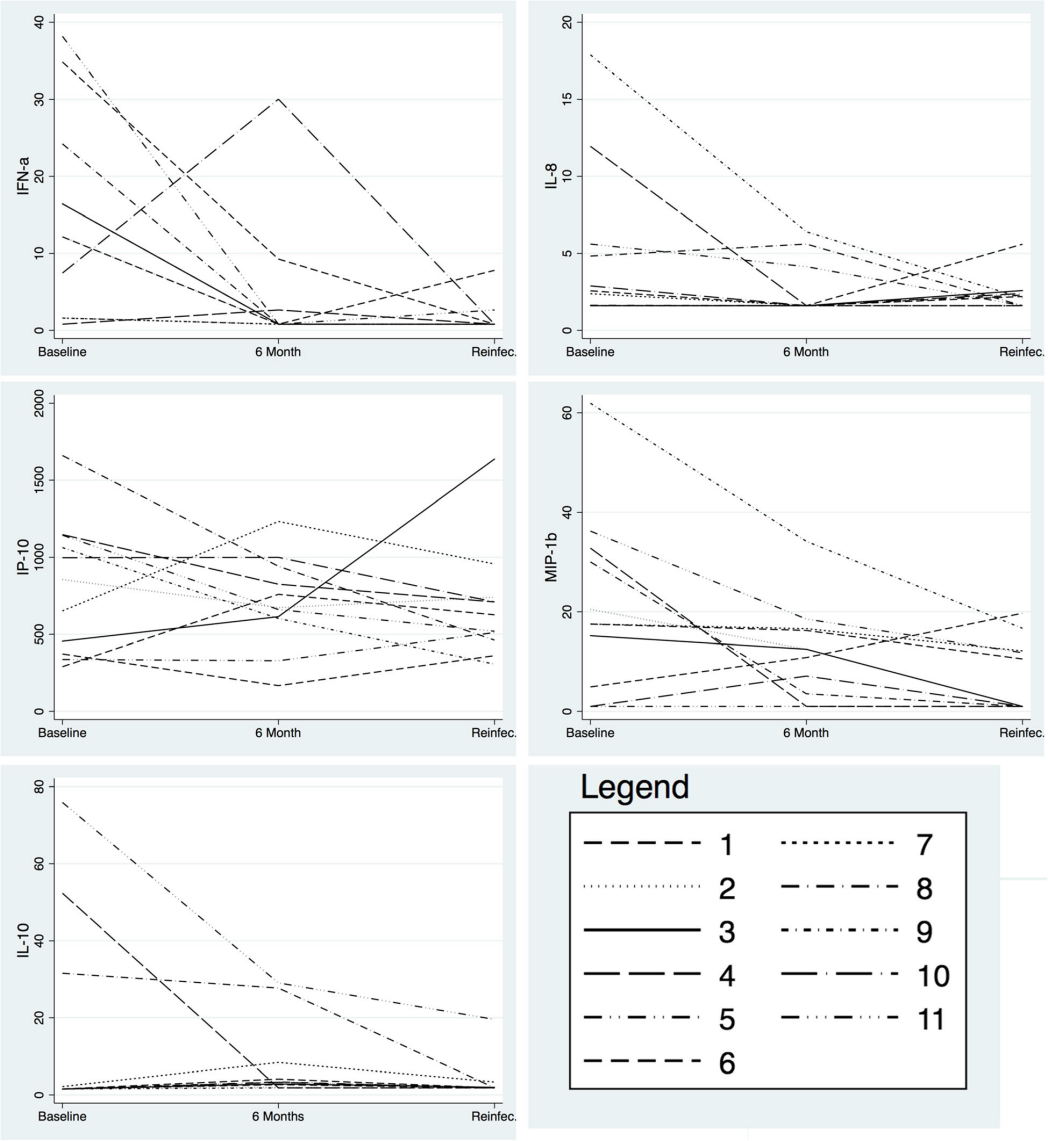
Selected chemo- and cytokine profiles (in pg/ml) in 11 participants with reinfections during study follow up. Participant number 11 was the only individual whose baseline infection was an initial infection

For 10 of these individuals, this represented at least their third episode of syphilis (Table 1). The 11th individual was the only one to present with an initial episode of syphilis. His first episode of syphilis was characterized by a diffuse maculopapular rash and a RPR titre of 1/32. He had tested TPA and RPR negative 6 months prior. He received 2.4MU BPG via intramuscular injection and his RPR titre declined to 1/4 at 3 months. At nine months following condomless sex, he presented with a less pronounced maculopapular rash and an RPR titre rise to 1/32. Of the 11 individuals, his baseline syphilis contained the highest or second highest levels of IL-10, IFNα, CCL4 and IP-10 (Participant 11, Fig. 1). At the time of his reinfection his cytokine levels did not attain the levels of his initial infection (Fig. 1).

### Paired comparisons of cytokines

Comparing to their baseline infections, paired analyses of the 11 individuals with reinfections during follow-up revealed that these reinfections had lower concentrations of IFNα (0.8 [IQR 0.8–0.8] vs. 12.2 [IQR 1.6–24.2], *P* = 0.004) and CCL4 (0.9 [IQR 0.9–12.2 vs. 17.5 [IQR 4.9–32.8], *P* = 0.022]; Table 2). These associations remained after excluding the individual whose baseline infection was his first (initial) episode of syphilis. In general, there was a decline in cyto- and chemokines between baseline and 6-month visit with little or no increase at the time of reinfection (Fig. 1).

**Table 2:** Plasma cytokine and chemokine concentrations in baseline, 6-month follow-up and repeat episodes of syphilis (pg/ml)

	Baseline infection	6 month	Repeat infection	*P*-Value^a^
IFNα	12.1 (1.6–24.2)	0.8 (0.8–2.6)	0.8 (0.8–0.8)	0.004
IFNγ	1.5 (1.5–8.9)	1.7 (1.5–10.3)	1.54 (1.5–10.6)	0.714
IL1β	1.5 (1.5–1.5)	1.57 (1.5–1.5)	1.57 (1.5–1.5)	.
IL12p40	1.0 (1.0–1.0)	1.0 (1.0–1.0)	1.0 (1.0–1.0)	.
IL12p70	1.6 (1.6–1.6)	1.6 (1.6–1.6)	1.6 (1.6–1.6)	.
IP-10	855.4 (372.7–1143.6)	672.9 (602.6–941.6)	626.3 (462.0–740.4)	0.424
MCP-1	242.1 (212.8–303.1)	278.37(259.2–312.2)	260.7 (225.8–294.0)	0.533
CCL3	1.6 (1.6–4.9)	1.6 (1.6–1.6)	1.6 (1.6–1.6)	0.127
CCL4	17.5 (4.9–32.8)	12.4 (3.5–16.6)	0.9 (0.9–12.1)	0.022
IL-4	1.6 (1.6–1.6)	1.6 (1.6–1.6)	1.6 (1.6–1.6)	.
IL-5	1.6 (1.6–1.6)	1.6 (1.6–1.6)	1.6 (1.6–1.6)	.
IL-6	1.3 (1.3–1.3)	1.3 (1.3–1.3)	1.3 (1.3–1.3)	.
IL-7	1.1 (1.1–1.6)	1.1 (1.1–1.1)	1.1 (1.1–1.1)	.
IL-8	2.5 (1.6–5.6)	1.6 (1.6–4.1)	2.1 (1.6–2.4)	0.265
IL-10	1.5 (1.5–31.5)	3.3 (1.9–8.4)	1.8 (1.8–1.9)	0.787
IL-17A	1.5 (1.5–1.5)	1.5 (1.5–1.5)	1.5 (1.5–1.5)	0.489
RPR titre	128 (64–128)	8 (2–16)	32 (16–128)	0.07

All values are median and interquartile range

^a^ P-value is for paired comparison of reinfections with baseline infections (Wilcoxon’s signed rank test)

### Correlation between number of prior episodes of syphilis and individual cyto/chemokines

There was a weak negative association between IFNα and the number of episodes of syphilis (Rho = − 0.42; *P* = 0.050; Table 3).

**Table 3:** Spearman’s correlations between plasma cytokine and chemokine concentrations at the time of syphilis diagnosis and number of prior diagnoses of syphilis (baseline and repeat infections combined, *N* = 22)

	Spearman’s correlation	*P*-Value
IFNα	−0.42	0.050
IFNγ	−0.07	0.761
IL1β	–	–
IL12p40	–	–
IL12p70	0.17	0.42
IP-10	−0.22	0.327
MCP-1	0.141	0.531
CCL3	−0.09	0.697
CCL4	−0.27	0.222
IL-4	–	–
IL-5	–	–
IL-6	0.17	0.428
IL-7	0.14	0.532
IL-8	−0.31	0.156
IL-10	−0.04	0.84
IL-17A	−0.28	0.202

## Discussion

A diagnosis of syphilis in humans and animal models is typically characterized by elevations in both plasma pro-inflammatory (TNFα, IFNγ, CCL4, IP-10) and anti-inflammatory cytokines (IL-10) [11–18]. This immunological profile however varies according to a number of parameters including stage of syphilis [4, 17–20], HIV-infection status [20] and the number of previous episodes of syphilis [4, 21, 22]. Our study is the first that we are aware of that has been able to assess the immune profile of the same individuals at the time of reinfection.

Our study is weakened by the small number included and the fact that only one of the 11 baseline episodes of syphilis was a confirmed initial episode of syphilis. Larger studies would be required to be able to conduct multivariable analyses to control for the role of confounders such as stage of syphilis, number of episodes of syphilis, time between episodes of syphilis and HIV status. With a sample size of only 11 we deemed the risk of type II errors too large to warrant conducting multivariable analyses. As a result we cannot exclude the possibility that our results are due to confounding. Nonetheless our results are commensurate with the thesis that repeat syphilis presents with an attenuated immune response. The individual with an initial syphilis at study inclusion had a more pronounced elevation in IL-10, IFNα, CCL4 and IP-10 than the individuals with repeat syphilis. His second episode of syphilis was also clinically less dramatic and characterized by little or no elevation in plasma cytokines (compared to his 6-month levels). Furthermore, shortly after completing the 24-month follow-up period (and therefore not included in this study) this individual had a third episode of syphilis which was asymptomatic.

In the paired comparisons, plasma IFNα and CCL4 were lower at the time of the reinfection. The fact that repeat episodes had lower IFNα and CCL4 even after excluding the only individual whose baseline syphilis represented an initial infection suggests that each additional episode of syphilis results in a more attenuated immune response than the previous episode. As noted above, this finding is weakened by the fact that in 10 out of 11 individuals the baseline episode of syphilis was not the individual’s first episode of syphilis. What we are comparing is earlier with later episodes of syphilis in the same individual. Our findings of lower plasma IFNα and CCL4 in later episodes of syphilis is commensurate with immunological and clinical findings from other studies. Previous studies have, for example, found that each subsequent diagnosis of syphilis is associated with a step-wise increase in peak RPR at the time of diagnosis, a faster decay in RPR but then a higher nadir RPR result following the episode of syphilis [4, 21, 22]. One of these studies found that the RPR result did not serorevert in any individuals with four or more episodes of syphilis [4]. A recent study has found a stepwise increase in the probability that syphilis presents asymptomatically with increasing numbers of episodes of syphilis [3]. These findings suggest that individuals with multiple episodes of syphilis may develop a degree of immunity to infection by *T. pallidum* [1] and that each episode of syphilis has an additive effect in the development of immunity. Further studies are required to assess if the attenuated immune response is correlated with a lower bacterial load and hence infectivity. This would be relevant for studies modeling syphilis spread and might provide clues of use for *Treponema pallidum* vaccine design.

In a similar vein, our study builds on the findings of other contemporary studies which suggest a need to rethink the syphilis staging system used in settings such as ours where repeat syphilis constitutes an increasing proportion of all syphilis infections [1]. In a study of individuals with four or more episodes of syphilis from our center, for example, we found a step-wise increase in the proportion diagnosed with latent (asymptomatic) syphilis with increasing numbers of episodes of syphilis [3]. Similarly, in this study we found that 7 of the 11 episodes of repeat syphilis were latent syphilis. This classification was based on the fact that these 7 episodes were asymptomatic. The traditional staging system of syphilis typically depicts syphilis as progressing from primary to secondary and then early and late latent syphilis [23]. A large proportion of our patients with repeat syphilis do not develop symptoms of syphilis and thus classified as latent syphilis. This is likely due to the attenuated immune response to repeat syphilis and not reflective of a longer duration of infection as would be the case in initial episodes of latent syphilis. Future studies could explore this issue further by assessing if there are clinical, immunological, pathogen and pathological differences between individuals with early and late latent syphilis diagnosed during a first compared to a fourth (or higher) episode of syphilis. Consideration could be given to classifying latent syphilis in individuals with multiple previous episodes of syphilis as latent-repeat syphilis [1].

Unfortunately we were unable to sequence *T. pallidum* DNA from any of the episodes of syphilis included. We were thus unable to establish genotypically if the repeat syphilis was due to reinfection or reactivation as has been done in other studies [24, 25]. Given the high rates of partner change, low rates of condom usage and high syphilis incidence in this cohort [4] it is however likely that most if not all the repeat episodes are due to reinfections. We cannot, however, exclude the possibility that the episodes of repeat syphilis are due to reactivation rather than reinfection. Relapse syphilis may be characterized by a different immunological response to reinfection syphilis and this may influence our results. This misclassification bias would however be expected to bias the results towards the null-hypothesis.

## Conclusions

This study makes a small contribution to the growing evidence that the clinical and immunological presentation of repeat syphilis is more attenuated than initial syphilis. This is of considerable clinical and public health consequence. It is important that both clinicians and patients are aware of this as it provides extra motivation for periodic serological screening in persons who have had a diagnosis of syphilis. These individuals are not only at a considerably higher risk of syphilis than the general population, their subsequent syphilis is also more likely to be asymptomatic and serological screening may be the only way to diagnose this syphilis episode in a timeous fashion.

## Abbreviations

CCL: Chemokine ligand
IFN: Interferon
IL: Interleukin
IQR: Interquartile range
MIP: Macrophage inflammatory protein
MSM: Men who have sex with men
PCR: Polymerase chain reaction
RPR: Rapid plasma reagin
TNF: Tumour necrosis factor
USA: United States of America
